# 52. Mycophenolate for concurrent macrophage activation syndrome and lupus flare: a case report

**DOI:** 10.1093/rap/rky034.015

**Published:** 2018-09-20

**Authors:** Andrew Porter, Gagandeep Takhar, Venkat Reddy

**Affiliations:** Rheumatology, West Middlesex Hospital, London, UNITED KINGDOM

**Introduction:** Macrophage activation syndrome (MAS) is a rare but life-threatening condition characterised by dysfunctional macrophage activation leading to overproduction of cytokines and phagocytosis of erythrocytes, leucocytes and platelets. Due to its mimicry of other conditions it is often under recognised leading to significant delays in diagnosis. We describe a case of a 42 year old lady with active systemic lupus erythematosus (SLE) who presented with lethargy, rash and joint pain and within 3 days of onset of significant cytopenias was diagnosed with MAS. This early diagnosis meant that her condition responded and resolved with less intensive immunosuppression.

**Case description:** A 42 year old lady who originally presented aged 32 with fevers, fatigue and cervical lymphadenopathy. Lymph node biopsy demonstrated a necrotising lymphadenitis consistent with Kikuchi’s disease. She received symptomatic treatment and her symptoms resolved. Aged 37 she developed lymphadenopathy, fever, malaise as well as a scaly erythematous rash initially affecting her cheeks but then spreading to involve her chest and upper arms. In addition, she was experiencing generalised joint pains, morning stiffness, a low-grade fever and alopecia. Initial investigations showed pancytopenia with a raised ESR, positive ANA (1:160) and anti-dsDNA antibodies (149IU/ml), positive anti-Ro, anti- RNP and anti-SM antibodies based on which she was diagnosed with SLE. She was commenced on treatment with a reducing dose regimen of prednisolone as well as hydroxychloroquine 200mg twice a day with significant improvement in her symptoms. On reducing the prednisolone dose she found her symptoms worsened, particularly with regards to joint pains and rash. She was therefore commenced on treatment with azathioprine 50mg daily and remained well. She was keen to reduce the amount of medication she was taking so stopped azathioprine. She was admitted to hospital in December 2017 with right sided neck and jaw pain along with swelling over the parotid region. Ultrasound examination findings revealed bilateral parotitis. She was treated with clindamycin for suspected infective parotitis and her symptoms resolved. A week later she developed an erythematous scaly rash, initially affecting her face but then spread to cover her whole body. Histological findings of the skin biopsy were suggestive of a drug rash. However, laboratory parameters revealed elevated anti-dsDNA antibodies (276IU/ml) and low complement (C3 0.47 g/L, C4 0.08 g/L). Taken together, these findings were indicative of a flare up of SLE. Hence, she was treated with a higher dose of oral prednisolone of 40mg/day concomitantly with a dose of intravenous Methylprednisolone 500mg attaining good response. In early 2018 she became more unwell with recurrence of the rash, lethargy, back pain, nausea and worsening parotid pain. Blood tests showed pancytopenia (WCC 3.1, platelets 131, haemoglobin 87d/L), with a raised LDH (592U/L), Ferritin 3300 ug/L, triglycerides 3.81mmol/L. CT of chest, abdomen and pelvis revealed no lymphadenopathy. A bone marrow trephine biopsy demonstrated atypical/dysplastic features and haemophagocytosis. Collectively, these findings were indicative of macrophage activation syndrome and she was treated with a single dose of intravenous methylprednisolone 1g and commenced on mycophenolate mofetil 1g twice a day with reducing dose regimen of oral prednisolone. She was monitored frequently for signs of flare up of lupus and to evaluate for MAS. Laboratory parameters were also measured daily for a week and subsequently, weekly. There was a significant improvement in her symptoms consistently. Over a period of five weeks, the laboratory markers including ferritin, LDH, white cell count, platelets and haemoglobin normalised. At six months since concurrent diagnosis of MAS and lupus flare, she remains well on a combination of mycophenolate 1g twice daily and oral prednisolone of 10 mg/day.

**Discussion:** Concurrent MAS and flare up of lupus has been previously described and MAS may be the presenting feature of SLE. However, clinical features and laboratory indices may not clearly distinguish lupus flare from MAS, which is associated with high mortality. Therefore, a combination of potent immunosuppressants is used to treat concurrent MAS and lupus flare. In our patient with SLE described here, a high index of suspicion of MAS led us to obtain appropriate investigations without delay. Thus, securing an early diagnosis and treatment. We hypothesised that active lupus manifesting in skin and parotid glands was triggered by T cell infiltration and seemed to respond favourably to relatively low dose corticosteroid therapy. Ciclosporin and other immunosuppressive agents such as etoposide, cyclophosphamide, rituximab and plasma exchange have been used effectively to treat MAS in the context of Lupus. Treatment with ciclosporin is associated with significant adverse effect profile. Therefore, alternative immunosuppressive regimen with mycophenolate was considered in combination with low to moderate dose of oral corticosteroids. We monitored the clinical features and laboratory parameters regularly. We used the trend of changes in laboratory parameters to guide treatment decisions. As previously reported, we found that it may take weeks for the laboratory parameters to normalise whereas the trend of the kinetics of biochemical parameters was evident at shorter time interval and is likely influenced by the activation status of macrophages perhaps reflecting their role in lipid metabolism. Over a period of five weeks from the first dose of IVMP and mycophenolate, we noted significant improvement in clinical symptoms, signs and normalisation of laboratory parameters, sustained at six months follow-up. To our knowledge, there exists only one previous case report where mycophenolate alone in combination with relatively modest dose of corticosteroids has been used to treat MAS in a patient with lupus.

**Key Learning Points:** Macrophage activation syndrome is a rare but life-threatening complication of autoimmune rheumatic disorders such as SLE. Clinical features of macrophage activation syndrome and lupus flare may be indistinguishable. A high index of suspicion is key to attaining early diagnosis. Early diagnosis and judicious monitoring may help manage MAS and lupus flare effectively with mycophenolate and corticosteroids.

**Disclosure: A. Porter:** None. **G. Takhar:** None. **V. Reddy:** None.

